# Measurement of Quality of Life IV. Use of the SEQOL, QOL5, QOL1, and Other Global and Generic Questionnaires

**DOI:** 10.1100/tsw.2003.78

**Published:** 2003-10-13

**Authors:** Soren Ventegodt, Joav Merrick, Niels Jorgen Andersen

**Affiliations:** The Quality of Life Research Center, Copenhagen K, Denmark

**Keywords:** Quality of Life, QOL, SEQOL, QOL5, QOL1, measurement, human development, holistic medicine, public health, Denmark

## Abstract

Quality-of-life (QOL) rating scales can be used to measure and describe the quality of life of a specific population or patient group. Many decisions can be taken and policies implemented when we know more about a group or population. The global quality-of-life concept may help in expressing the objective of the initiatives taken to benefit specific groups. The objective may be that we hope the efforts will increase their quality of life by a certain percentage. This explicit expectation will force the decision makers to stand by their noble intentions. They are obliged to evaluate their efforts and will have to learn something from it.A questionnaire thus constitutes a useful scientific instrument, as databases based on comprehensive and thorough questionnaire surveys that seek to encompass all aspects of life can provide valuable and precise information. The value of such a database depends on the correct use of the questionnaires and this paper examines some examples of how quality-of-life rating scales can be used.We identified at least ten ways to use the quality-of-life questionnaire: describing the quality of life of a population or patient group; formulating an objective for support, treatment, or care; screening or identifying individuals who need treatment; evaluating treatment and care; facilitating communication between physician and patients; involving the patient in the decision-making process; allocating resources; investigating the causal relation between the quality of life and ill health in prospective studies; creating an awareness of the quality of life and health promotion; and helping the practitioner to accumulate knowledge.Enhancing the quality of life is therefore a determining factor in the process of increasing awareness and responsible conduct in relation to the environment, natural resources, the working environment, and the structure of society. Putting the quality of life on the agenda inherently has a constructive and positive effect on the life and functioning of the individual and society.

## INTRODUCTION

Quality-of-life (QOL) rating scales can be used to measure and describe the quality of life of a specific population or patient group. Many decisions can be taken and policies implemented when we know, as an example, what kind of life people with migraine or unemployed people above the age of 50 years have. We might wish to consider a particularly vulnerable group of people. The quality-of-life concept may help in expressing the objective of the initiatives taken to benefit specific groups. The objective may be that we hope the efforts will increase their quality of life by a certain percentage. This explicit expectation will force the decision makers to stand by their noble intentions. They are obliged to evaluate their efforts and will have to learn something from it.

Quality-of-life ratings can therefore be used as a scientific gauge in controlled clinical trials. The physician assesses how the person functions after he or she has undergone medical intervention. The person then has to fill out a questionnaire stating how he or she feels and describe his or her quality of life in other ways[1]. When such questionnaires are used clinically, they stimulate conversation between the doctor and the patient. The ideal place for the patient to complete the questionnaire would be in private. The ensuing conversation will make patients take a closer than usual look at their life. This will make it easier for the person to change their lifestyle.

The questionnaire can likewise serve to open discussion when the patient is about to undergo treatment. The patient is often not made aware of the advantages and disadvantages of different treatments. This is because the physician believes that the patient knows enough about the various options (nontreatment vs. treatments A, B, etc.) and how the medical literature evaluates the options. The patient would be greatly helped in deciding what treatment is best for him or her if something was known about the quality of life of other people who have undergone similar treatments. Quality-of-life ratings can thus be used to give the patient greater involvement in the medical process.

Measuring the quality of life is also useful in scientifically assessing the relationship between the quality of life and the development of illness. It is difficult and somewhat vague to establish what is meant by feeling good and being ill. It is an in-between, gray zone of discomfort, dissatisfaction, and slight disability that is difficult to put into words and hence not very well understood. A quality-of-life scale enables a population group to be monitored for a number of years and psychosocial and other factors prior to illness to be established.

Questionnaires are generally the most cost-effective way to collect information. The cost effectiveness — that is, the cost per unit of useful information gained — of a thorough, self-administered questionnaire can be much higher than that of an unstructured medical consultation or a chemical/physical screening process such as a blood test. When a person expresses some indeterminate complaint about poor well being, a global and generic questionnaire on the quality of life like the SEQOL (self-evaluated quality of life) questionnaire [2], the QOL5 questionnaire[3], or even the QOL1 questionnaire[3] can provide 100 to 1,000 times more relevant information about the condition of the person than a standard blood test. Moreover, the blood test is significantly more expensive (about EURO/$20) than the questionnaire (about EURO/$5). Thus, the ratio between cost and information is at least 5,000 times higher for the questionnaire.

A questionnaire thus constitutes a useful scientific instrument, as databases based on comprehensive and thorough questionnaire surveys that seek to encompass all aspects of life can provide valuable and precise information. The value of such a database depends on the correct use of the questionnaires for suitable purposes[4,5]. This paper examines some examples of how quality-of-life rating scales can be used (see Table 1).

**TABLE 1**
**Ten Ways to Use the Questionnaire**

1. Describing the quality of life of a population or patient group
2. Formulating an objective for support, treatment, or care
3. Screening/identifying individuals who need treatment
4. Evaluating treatment and care
5. Facilitating communication between physicians and patients
6. Involving the patient in the decision-making process
7. Allocating resources
8. Investigating the causal relation between the quality of life and ill health in prospective studies
9. Creating an awareness of the quality of life and health promotion
10. Helping the practitioner to accumulate knowledge

## DESCRIBING THE QUALITY OF LIFE OF A POPULATION OR GROUP

The questionnaire can be used to assess the quality of life of such population groups as people with chronic pains[6], people with skin diseases[7], or people who are unemployed, where each person cannot be interviewed individually. It can either be used on the whole group or on a randomly selected representative sample of the group. This will provide the researcher with a standardized rating for quality of life that can be compared with the quality-of-life rating of the population as a whole (see the reference values in Table 2) and/or with ratings of other population groups that have had their quality of life rated using the same questionnaire (note that the SEQOL questionnaire is generic and not aimed at any specific group of people[2]).

A questionnaire is thus an inexpensive and time-saving method of assessing a population. Further, if several surveys have been carried out using the same questionnaire, the researcher will eventually get a standardized and comparable rating scale. The insight gained by the analysis of a questionnaire is less intimate than, for example, when in-depth personal interviews are conducted. A survey based on a questionnaire should be understood as the first stage in gaining knowledge about a group of people, where the questionnaire is an effective method for this purpose.

An example could be the desire to assess the quality of life of people who have had a kidney transplant and compare this to the quality of life of the population in general. The database we have created can control all data (for example, by simple reference checks in our collection of statistical tables) for age, gender, geographical location, etc. to obtain a better picture of the quality of life of the group in question.

## FORMULATING AN OBJECTIVE FOR SUPPORT, TREATMENT, OR CARE

Imagine a situation in which a group of ill people or a vulnerable social group needs some form of support, but you are unsure as to what specific support would be most effective. A good starting point would be to make enhancing the quality of life the main objective. This could then be defined in more specific terms, for example, improving how satisfied people are with their jobs or their family, improving their situation such that people feel that their needs are being fulfilled more effectively, or helping them to experience greater meaning in life.

**TABLE 2**

| **Quality of Life in Denmark: Population Mean (%)** | **18–88 Years (Population-Based Study)** | | | **31–33 Years (a Cohort)** | | |
|---|---|---|---|---|---|---|
| | **Women** | **Men** | **Both Sexes** | **Women** | **Men** | **Both Sexes** |
| Well being | 71.6 | 72.5 | 72.0 | 71.7 | 72.2 | 71.9 |
| Satisfaction with life | 69.4 | 69.5 | 69.5 | 69.6 | 69.1 | 69.3 |
| Happiness | 65.8 | 65.2 | 65.5 | 66.6 | 65.6 | 66.2 |
| Fulfillment of needs | 69.8 | 68.9 | 69.3 | 69.4 | 67.1 | 68.3 |
| Family, work, and leisure time | 75.9 | 76.0 | 76.0 | 75.4 | 74.4 | 74.9 |
| Satisfaction with relationships | 69.6 | 68.9 | 69.3 | 67.9 | 66.2 | 67.1 |
| Realization of life potential | 44.8 | 45.2 | 45.0 | 46.4 | 44.9 | 45.7 |
| Objective factors | 69.1 | 69.3 | 69.2 | 73.5 | 71.2 | 72.4 |
| **Overall quality of life (SEQOL)** | 66.8 | 67.3 | 67.1 | 69.0 | 67.6 | 68.3 |

*Note:* Readers who want to measure the quality of life of a particular group of people can use the population values presented here for comparison. For a difference between the studied group's mean quality of life and the population means to be statistically significant ($p < 0.05$), the group being studied must have at least as many people as given by the sensitivity[2], depending on the size of the difference. Significance can be improved through the use of a large reference group and exact calculations.

It is generally easy to set ambitious goals for a specific intervention. However, it is more difficult to determine whether those goals are being met. If the goals are expressed in terms of the concepts presented in a questionnaire, then the monitoring process becomes easier. The questionnaire can be distributed before and after the intervention as well as on a continuing basis if the action taken is to be an on-going process lasting several years.

If the questionnaire surveys do not show any increase in the quality of life of the target group, the objective of the intervention is not being met and the action should be reassessed. Explicit demands for assessing progress and analyzing the value of social or medical investment are commonplace and considered necessary in other areas of society. No private company would invest large sums of money in a product or a service without some assurance that the customer and the market (and thereby the company itself) will consider the product or service worth buying. But this is not the case with the public sector, which directs large amounts of money towards social and labor market initiatives.

The most common methods used for evaluating the effectiveness and efficiency of medical and social services often lack direct input from the users of the services. A questionnaire on the quality of life is a very direct approach and should be able to supply the researchers with interesting indicators as to the results of the efforts. The questionnaire and the direct feedback it provides force professionals in the public care system and the relevant decision makers to commit themselves to revising the action taken if the original objectives are not reached.

A questionnaire that gives quantitative answers will furthermore encourage the decision makers to formulate their targets more accurately. For example: the public authorities decide to aim to improve the quality of life by 20% for a specific group of workers with many back problems (corresponding to a change from, for example, neither good nor poor to good). To reach this goal, the work routine has to be modified in some way. The parties involved can agree in advance that if the improvement is less than anticipated (demonstrated by the questionnaire), certain predetermined changes should be made (these changes do not necessarily involve increased effort; readjustment is more appropriate in some cases). In the absence of well-defined objectives and agreements as to what should be done, if the target is not attained, most initiatives simply continue until the money is spent and the project ends — often without any notable improvement in the quality of life of the target group and without the financing authority having learned anything about allocating resources.

## SCREENING/IDENTIFYING INDIVIDUALS IN NEED OF TREATMENT

If, like the present health services, we accept the value of screening, then the distribution of a quality-of-life questionnaire could be an extremely useful aid to this process. Screening enables individuals who need treatment to be identified more rapidly than if they developed the symptoms that would motivate them to visit a doctor. Our investigations showed that a low quality of life is a broad indicator of a need for support. If the questionnaire is mailed to a social group that is recognized to be vulnerable (for example, single mothers with more than one child or men in their fifties unemployed for a long time), then a low rating on a quality-of-life scale, difficulties caused by illness, or other self-reported problems could indicate that specific forms of support or treatment would be appropriate.

Screening has advantages and disadvantages from a quality-of-life point of view. Screening does facilitate the identification of individuals with an unrecognized need for treatment. However, since the survey cannot be carried out ethically without informing people as to its objective, screening tends to spread anxiety and supports people's negative expectations towards life in a way that does not improve their quality of life (as the debate in Denmark about mammography has shown).

The authoritarianism inherent in the very idea of screening ("We are going to examine you and decide whether you are part of a high-risk group") hampers the individual's confidence in and knowledge about him- or herself. This is unfortunate, as mobilizing these personal resources is crucial in the attempt to improve the quality of life.

Nevertheless, if the health authorities wish to screen for a particular disease, a questionnaire about the quality of life would be less frightening or confidence shaking than a physical examination in which the patient is confronted by radiation, tubes, cold metal, and other intimidating medical devices. The questionnaire asks people to relate how they get on at work, whether they are happy with their sex lives, whether they experience meaning in life, and what values they regard as most important. Such questions put people's health and way of life on the agenda constructively and invite contemplation of lifestyle and perhaps a positive consideration of how changes could take place. This method contrasts sharply with the threatening warnings patients receive in the traditional medical screening of specific diseases ("Change your way of life, or else...!") and with the terrible feeling of being powerless and entirely dependent on the doctor's verdict and treatment. If the starting point is the quality of life, people will not as easily experience a feeling of being at the mercy of external forces but will, instead, be encouraged in their belief that they themselves are capable of controlling their lives and that they are responsible for their lives.

## EVALUATING TREATMENT AND CARE

Quality of life is being used more and more as an indicator of the effect of a treatment in controlled clinical trials. Narrow aims for treatment may be relevant, but when the ultimate aim of any treatment, support, or care is to improve a person's quality of life, the quality of life should be measured globally. Numerous questionnaires are available from the scientific literature[8,9,10] and our newly developed rating scales can be used without difficulty.

If a quality-of-life rating is used for this purpose, it may be discovered that the treatment already prescribed has very little effect on the rating of the quality of life. The evaluation of quality is a demanding task, because the process requires critical scrutiny of the procedure used and may require substantial changes in the usual procedure when the targets for treatment or care are not achieved.

Any evaluation of quality requires the formulation of a set of predetermined goals. It is never sufficient to say: "Let's see what kind of improvement occurs", because this attitude removes all drive and purpose from quality evaluation. Ambitious and explicit objectives are necessary, after all, we know what we want the treatment to accomplish.

It is quite straightforward to take a group of people, who are doing poorly and try to make them feel 10, 20, or 40% better in a sense to be defined (for example, well being) and then to ascertain whether the group or the individual has attained this improvement at the end of treatment. The difficult part lies in acknowledging that the quality of life is a phenomenon with so many facets all deeply rooted in a person's life that substantial changes are required to achieve any real improvement. It is therefore important to start by setting modest targets for improving the quality of life and to remember that people themselves can make the most significant changes in or improvements to their lives.

## FACILITATING COMMUNICATION BETWEEN PHYSICIAN AND PATIENTS

Pilot surveys have shown that individuals who have filled out the quality-of-life questionnaire before a medical consultation are more motivated to embark on a discussion about issues related to values, lifestyle, health, sickness, and so on. General practitioners may therefore use the questionnaire to prepare patients for the discussion. One of the aims of this conversation is to help the patient to achieve a healthier way of life.

People who spend an hour considering their lifestyle before arriving at the office or clinic of the physician can better help the physician in formulating a more accurate diagnosis by contributing more precise information about the illness. The physician can more easily discuss required changes in lifestyle. This approach can also reduce or eliminate the person's need for symptom-suppressing drugs, and the doctor may thus not need to prescribe them.

At the Quality of Life Research Center in Copenhagen, we are currently developing an electronic version of the questionnaire that can help both the patient and the physician in discovering the existential weaknesses that appear to be connected to illness and other health-related problems.

## INVOLVING THE PATIENT IN THE DECISION-MAKING PROCESS

When people are facing major surgery, they can rarely predict the consequences of treatment such that they can make a qualified decision between alternatives. This is one reason why people are not typically involved in major decisions on medical treatment.

Doctors may not have any systematic knowledge of the consequences either. Some types of treatment make the patient feel worse, but prolong life. Another type of treatment may make the patient feel better subjectively, but his or her existential quality of life experience will be reduced significantly, as a consequence of alienation or resignation. A third type of treatment may prolong life and improve certain life functions but lead to deterioration in others, for example, sexual or mental performance. A systematic assessment of treatments and their consequences would enable people to compare the different treatments and choose the ones most acceptable to them[11].

How can we assess the various treatments and their consequences and achieve a broad overview? Years of experience provide a physician with a certain overview, but this does not adequately aid the patient in the decision-making process. For the reasons mentioned above, it is relevant to assess the effect of treatment by the global quality of life, for example, with the help of our questionnaire. If the quality of life of a representative number of people is assessed after they have undergone different types of treatment, this will produce a set of comparable numbers that, due to their simple interpretation, should appeal to every patient facing the same choice between different treatments. Giving people the opportunity to assess the alternatives is the first step required to incorporate them into the medical decision-making process.

Formulating the results of treatment and effect in terms of the quality of life is a great advantage compared with the rigid and often technical assessment of function; everyone can relate to the quality of life.

## ALLOCATING RESOURCES

When the allocation of resources is politically or administratively determined, it is useful to focus on the objectives of using the resources. One of the main objectives should be to increase the quality of life of the population. It is therefore not justifiable to invest large sums in high-tech equipment and new expensive drugs without some guarantee that the investment made will improve the quality of life.

The medical community has expressed a firm conviction for years that advanced technology and new sophisticated drugs provide inherent benefits the users. This belief, however, has only been partly confirmed by clinical experience in the last few years. It has, for example, not been conclusively verified through empirical studies that electronic fetal monitoring leads to better results than those produced by trained midwives. Still, this equipment is now standard in most modern maternity wards.

All expensive, labor-intensive, high-tech innovations in treatment and care should be evaluated critically as to the effects on the patient's quality of life. This must be done to ensure that technological progress does not take place for its own sake; it must satisfy human needs. The dimension of the quality of life can serve an important corrective function here. Healthcare is very expensive, and it is therefore in the interest of society to ensure that money is used to benefit the patients rather than the many medico-technical and pharmaceutical companies.

Nevertheless, laypeople's perceived quality of life must comprise the basis of care and treatment. The much-debated concept of quality-adjusted life years (QALY) is not an adequate basis for politicians to set priorities in the healthcare sector.

The ideal situation is one in which people themselves choose among the various treatments available. Laypeople are probably far less interested in being subjected to high-tech equipment than doctors and the producers of high-tech equipment would like them to be. If the objective is to position people at the center of the decision-making process with a responsibility for their own lives, quality-of-life questionnaires would be useful and appropriate instruments.

## INVESTIGATING THE CAUSAL RELATION BETWEEN QUALITY OF LIFE AND ILL HEALTH IN PROSPECTIVE STUDIES

Actual illness or disease is preceded by a period of feeling unwell, poor well being, and other health problems. This gray zone has not been properly investigated and understood; one reason is that the concepts and the scientific instruments required for this area are lacking. A quality-of-life rating scale can be used to follow a population prospectively and to investigate a plausible connection between a low quality of life and the development of illness or disease.

Thus, if the individual's journey through the entire gray zone from good health to illness or from illness back to a recovery can be traced, this can improve understanding of the quality of life and other subtle factors that influence the development of illness but are still conceptually and terminologically vague. How do we understand illness as a dynamic entity that develops gradually as a function of our way of life — and in what sense and to what degree can we control and take responsibility for illness and health?

The concept of the quality of life may bridge the biological understanding of the human being and the existential and psychological aspects, which deals with experience and consciousness[12,13,14]. The future perspective then becomes a deeper understanding of the psychosocial contribution to the development of illness and a corresponding expansion of the theoretical foundation of medical science.

## CREATING AN AWARENESS OF QUALITY OF LIFE AND HEALTH PROMOTION

Responding to the questionnaire gives the respondents the opportunity to consider their own lives and their quality of life in depth. The process invariably triggers thoughts about the quality of life that, in turn, lead to increased consciousness about their own life.

Filling out a questionnaire can be a very fruitful exercise when this kind of consciousness is needed: for example, in an organization that desires to make employees more aware of issues related to the quality of working life. The process will have a further effect if the quality of life and the distribution of the questionnaires are put on the agenda at staff meetings, courses, etc. It is our experience that many people fill out the questionnaire voluntarily, because they think it is fun.

Awareness or consciousness about the quality of life inherently increases individuals' opportunities to take responsibility for their own lives and to tackle difficult situations well. In conjunction with this, people with a high quality of life, all other things being equal, are better equipped to make decisions in the world in which they live. The quality of life thus becomes a qualification for decision makers in politics, business, and organizations. Enhancing the quality of life is therefore a determining factor in the process of increasing awareness and responsible conduct in relation to the environment, natural resources, the working environment, and the structure of society. Putting the quality of life on the agenda inherently has a constructive and positive effect on the life and functioning of the individual and society.

## HELPING THE PRACTITIONER TO ACCUMULATE KNOWLEDGE

The use of a global quality-of-life assessment tool can help practitioners to develop theoretical models of quality of life in practice. This might be a very important issue, as too many physicians never stop and reflect deeply enough on what really helps their patients. In this critical initial phase of global quality-of-life research, we need physicians and nurses to accumulate all their qualitative knowledge on quality of life and development of disease, and present it for the scientific community. So much important knowledge on the connection between quality of life and disease is lost with each generation of physicians, because wise physicians die without reporting their essential findings on the “soft issues” in their clinic.

## CONCLUSION

Global quality-of-life measures are estimated to be 5,000 times as cost effective in collecting information on a patient’s quality of life, health, and ability of functioning, as an unstructured medical consultation or a chemical/physical screening process such as a blood test. The new generation of generic and global quality-of-life questionnaires like SEQOL, SCREENQOL, QOL5, and QOL1 are a source of cheap and reliable information on any group of patients or any population. Using the gained knowledge from a population screening, politicians and experts in public health can gain a highly useful feedback on their well-intended initiatives, making politics and decision making not an art, but a true science.

The performance of a medical clinic or health center can easily be measured using a short version like QOL5, or even QOL1, to be answered and scored in only a few minutes. Any physician who wants to really know the value of his efforts on a group of patients can get the knowledge needed, at a cost so low that he barely will notice it. All it takes to evaluate the gain of 20 patients given a specific treatment is 40 copies of the QOL5 questionnaire and one hour of study. Knowing the price and circumstances as well as the quality-of-life outcome of two or more alternative treatments, it is easy for the patient to make his choice of treatment. As quality of life is the issue of highest interest to most patients, discussing quality of life seems to be a perfect opening to a meaningful and fruitful dialog about difficult existential issues. Screening the population for low quality of life might be the most efficient way to prevent disease and future poor functioning, saving society billions of EUROs or USD. It will also save people much unnecessary suffering. Awareness of your own responsibility for life and health is often increased when the person is asked to fill in a comprehensive questionnaire on the global quality of life. The limitation of using quantitative measures of quality of life is that even the best rating scale cannot substitute compassion and wisdom. The use of quality-adjusted life years (QALY) in medical decision making seems to be such an unfortunate use of the quantitative quality-of-life measure. This problem will be dealt with in another paper on quality-of-life methodology[15].

## ACKNOWLEDGMENTS

This study was supported by grants from The 1991 Pharmacy Foundation, as well as by supplementary grants from Goodwill-fonden, the JL-Foundation, E. Danielsen and Wife's Foundation, Emmerick Meyer's Trust, the Frimodt-Heineken Foundation, the Hede Nielsen Family Foundation, Petrus Andersens Fond, Wholesaler C.P. Frederiksens Study Trust, Else & Mogens Wedell-Wedellsborg's Foundation, and IMK Almene Fond. We gratefully acknowledge the critical scrutiny and expert linguistic assistance of Ib Ravn, Ph.D. The research was approved by the Copenhagen Scientific Ethical Committee under number (KF)V.100.2123/91.

## References

1. Ventegodt, S., Hilden, J., and Merrick, J. (2003) Measurement of quality of life I. A methodological framework. *TheScientificWorldJOURNAL* **3**, 950–961.
2. Ventegodt, S., Henneberg, E.W., Merrick, J., and Lindholt, J.S. (2003) Validation of two global and generic quality of life questionnaires for population screening: SCREENQOL & SEQOL. *TheScientificWorldJOURNAL* **3**, 412–421.
3. Lindholt, J.S., Ventegodt, S., and Henneberg, E.W. (2002) Development and validation of QOL5 for clinical databases. A short, global and generic questionnaire based on an integrated theory of life. *Eur. J. Surg.* **168,** 107–113.
4. Fitzpatrick, R., Fletcher, A., Gore, S., Jones, D., Spiegelhaler, D., and Cox, D. (1992) Quality of life measures in health care. I. Applications and issues in assessment. *BMJ* **305,** 1074–1077.
5. Fletcher, A., Gore, S., Jones, D., Fitzpatrick, R., Spiegelhaler, D., and Cox, D. (1992) Quality of life measures in health care. II. Design, analysis and interpretation. *BMJ* **305**, 1145–1148.
6. Ventegodt, S., Merrick, J., and Andersen, N.J. (2003) Quality of life as medicine. A pilot study of patients with chronic illness and pain. *TheScientificWorldJOURNAL* **3**, 520–532.
7. Burckhardt, C.S., Archenholz, B., and Bjelle, A. (1993) Quality of life of women with systemic lupus erythemastosus: a comparison with women with rheumatoid arthritis. *J. Rheumatol.* **20**, 977–981.
8. McDowell, I. and Newell, C. (1996). *Measuring Health: A Guide to Rating Scales and Questionnaires.* Oxford University Press, New York.
9. Kaasa, S. (1992) Measurement of quality of life in clinical trials. *Oncology* **49(4),** 288–294.
10. Hollandsworth, J.G. (1988) Evaluating the impact of medical treatment on the quality of life. A 5-year update. *Soc. Sci. Med.* **26(4),** 425–434.

11. Johnstone, M.J. (1988) Quality versus quantity of life: who should decide. *Aust. J. Adv. Nursing* **6(1),** 31–37.
12. Spiegel, D., Bloom, J.R., Kraemer, H.C., and Gottheil, E. (1989) Effect of psychosocial treatment on survival of patients with metastatic breast cancer. *Lancet* **14(2),** 888–891.
13. Siegel, B. (1986). *Love, Medicine and Miracles*. Harper and Row, New York.
14. Birkelund, M. and Dige, U. (1993) *Disobedient Cancer Patients*. Hovedland, Århus, Denmark. (Danish)
15. Ventegodt, S., Merrick, J., and Andersen, N.J. (2003) Measurement of quality of life VI. Quality-adjusted life years (QALY) is an unfortunate use of the quality-of-life concept. *TheScientificWorldJOURNAL* **3,** 1015–1019.

## BIOSKETCHES

**Søren Ventegodt, MD,** is the Director of the Quality of Life Research Center in Copenhagen, Denmark. He is also responsible for a Research Clinic for Holistic Medicine in Copenhagen and is a popular speaker throughout Scandinavia. He has published numerous scientific or popular articles and a number of books on holistic medicine, quality of life, and quality of working life. His most important scientific contributions are the comprehensive SEQOL questionnaire, the very short QOL5 questionnaire, the integrated quality-of-life (IQOL) theory, the holistic process theory, the life mission theory, and the Danish Quality of Life Research Survey, 1991–94 in cooperation with the University Hospital of Copenhagen and the late pediatric professor Bengt Zachau-Christiansen. E-mail: ventegodt@livskvalitet.org. Website: www.livskvalitet.org/

**Joav Merrick, MD, DMSc,** is Professor of Child Health and Human Development affiliated with the Zusman Child Development Center and Division of Community Health at the Ben Gurion University, Beer-Sheva, Israel and presently the Medical Director of the Division for Mental Retardation, Ministry of Social Affairs, Jerusalem and the Director of the National Institute of Child Health and Human Development. He has numerous publications in the field of child and human development, rehabilitation, intellectual disability, disability, health, welfare, abuse, advocacy and prevention. Dr. Merrick received the Peter Sabroe Child Award for outstanding work on behalf of Danish Children in 1985 and the International LEGO-Prize ("The Children's Nobel Prize") for an extraordinary contribution towards improvement in child welfare and well being in 1987. E-mail: jmerrick@internet-zahav.net. Website: www.nichd-israel.com

**Niels Jørgen Andersen, MSc,** Professor, Department of Innovation and Economic Organization, Norwegian School of Management. This department conducts research and provides teaching in central topics related to innovation, business development, management of global companies, business history, and economic organization. Research activities within the Department are related to four core subjects within the discipline: business history, cooperative organizations, business development and entrepreneurship, and finally studies of industries with a special focus on the electricity industry. He is also the dynamic chairman of the nonprofit organization Stiftelsen Holistisk Medisin Scandinavia, which aims to support the scientific development, research, and documentation of complementary and holistic medicine in Scandinavia. E-mail: niels.j.andersen@bi.no. Website: www.bi.no/users/fgl93013/
